# *TIRAP* Rs8177376, Rs611953, Rs3802814, and Rs8177374 Polymorphisms and Their Association with Cervical Cancer Phenotype and Prognosis

**DOI:** 10.3390/genes13081365

**Published:** 2022-07-29

**Authors:** Justina Bekampytė, Aistė Savukaitytė, Agnė Bartnykaitė, Rasa Ugenskienė, Eglė Žilienė, Arturas Inčiūra, Elona Juozaitytė

**Affiliations:** 1 Oncology Research Laboratory, Oncology Institute, Lithuanian University of Health Sciences, LT-50161 Kaunas, Lithuania; justina.bekampyte@lsmuni.lt (J.B.); agne.bartnykaite@lsmuni.lt (A.B.); rasa.ugenskiene@lsmuni.lt (R.U.); 2 Department of Genetics and Molecular Medicine, Lithuanian University of Health Sciences, LT-50161 Kaunas, Lithuania; 3 Oncology Institute, Lithuanian University of Health Sciences, LT-50161 Kaunas, Lithuania; egle.ziliene@lsmuni.lt (E.Ž.); arturas.inciura@lsmuni.lt (A.I.); elona.juozaityte@lsmuni.lt (E.J.)

**Keywords:** cervical cancer, toll-like receptors, *TIRAP*, polymorphisms, phenotype, survival, prognosis

## Abstract

Cervical cancer is one of the most common cancers in women worldwide, which is typically caused by human papillomavirus (HPV). Usually, the toll-like receptor (TLR) signaling pathways eliminate the virus from the organism, but in some cases, persistent infection may develop. Unfortunately, the mechanism of immune tolerance is still unclear. Therefore, this study aimed to analyze *TIRAP* rs8177376, rs611953, rs3802814, and rs8177374 polymorphisms and to identify their impact on cervical cancer phenotype and prognosis. This study included 172 cervical cancer patients. Genotyping was performed using the PCR-RFLP assay. Univariate and multivariate logistic regression and Cox′s regression models were applied for statistical analysis. The results revealed that older age at the time of diagnosis was statistically linked with the rs8177376 T allele (OR = 2.901, 95% Cl 1.750–4.808, *p* = 0.000) and the rs611953 G allele (OR = 3.258, 95% Cl 1.917–5.536, *p* = 0.000). Moreover, the T allele of rs8177376 (OR = 0.424, 95% Cl 0.220–0.816, *p* = 0.010) was found to be statistically associated with the lower tumor grade. Thus, *TIRAP* polymorphisms might be employed in the future as potential biomarkers for determining the phenotype and prognosis of cervical cancer.

## 1. Introduction

According to the World Health Organization (WHO), cervical cancer is the fourth most frequently diagnosed cancer and the fourth most common cause of cancer death in women worldwide. 604,000 new cancer cases were diagnosed, whereas 342,000 deaths were confirmed in 2020 [1]. Human papillomavirus (HPV), particularly HPV16 and HPV18 which are members of the high-risk group, is primarily responsible for the persistent infection that leads to cervical cancer. HPV infection is spontaneously eliminated by the immune system. However, in some cases, HPV evades immune attack through various pathways, resulting in the development of a latent infection that eventually leads to the initiation of cervical cancer [2]. Therefore, additional research is needed to understand the molecular mechanisms underlying cervical cancer and develop less expensive, quicker, easier-to-use biomarkers for cancer prognosis [3].

Toll-like receptors (TLRs) are the main components of the human immune system that recognize pathogen-associated molecular patterns (PAMPs) derived from pathogenic bacteria, fungus, parasites, or viruses, including HPV. In response to pathogens, TLRs trigger the activation of numerous transcription factors, such as nuclear factor-kappa B (NF-κB), interferon regulatory factor 5 (IRF5), and activator protein 1 (AP-1). These transcription factors then encourage the production of pro-inflammatory cytokines such as interleukins (such as IL-1 and IL-6), tumor necrosis factor-α (TNF-α), chemokines, and interferons. Pathogens are eliminated as a result of this downstream signaling cascade [4,5]. However, an acute reaction may progress to chronic inflammation if signal transduction is disrupted and becomes uncontrollable. The structural or functional changes brought on by genetic variations, such as single-nucleotide polymorphisms (SNPs) in the genes encoding proteins implicated in the immune system and TLR signaling pathways, may affect the capacity to respond to infections appropriately. As a result, a number of infectious diseases as well as inflammatory-related cancers, such as cervical cancer, may manifest [5,6].

The toll-interleukin-1 (TIR) domain-containing adapter protein (TIRAP), also known as myeloid differentiation factor 88 (MyD88) adaptor-like (MAL) protein, is a component of the intracellular TLR signaling pathways [6,7,8]. It is well known that TIRAP recruits MyD88 to cell surface TLRs, such as TLR2 and TLR4 [8]. Moreover, several studies suggest that TIRAP may be necessary for endosomal TLR signaling via TLR7 and TLR9 [9,10]. The *TIRAP* gene encoding TIRAP is located at chromosome 11q24.2 [6]. Among all the adapter proteins involved in the TLR signaling cascade, *TIRAP* is the most polymorphic, and any SNP present can affect the signal transduction [6,7,8]. Previous studies demonstrated that *TIRAP* polymorphisms were associated with inflammatory diseases, such as tuberculous meningitis [11] and sepsis-associated acute lung injury [12]. *TIRAP* polymorphisms were examined for their possible correlations with cancer risk (lymphoma [13], glioma [14], and NHL [15]), but none of these correlations have yet been confirmed. On the contrary, substantial correlation with the risk of colorectal cancer was found [16]. There is a lack of knowledge if cervical cancer may be associated with *TIRAP* gene polymorphisms. Since studies have shown that TLR4, TLR7, and TLR9 play a significant role in HPV recognition [2,17,18], understanding how genetic variations in *TIRAP* affect signal transduction in TLR pathways and their effects on cervical cancer is crucial.

To investigate the influence of *TIRAP* polymorphisms on cervical cancer, we decided to analyze four polymorphisms, rs8177376, rs611953, rs3802814, and rs8177374, that were chosen using the RefSNP Report database (dbSNP) based on their MAF and location in the gene. To our best knowledge, this is the first study analyzing the function of selected SNPs in cervical cancer, including their associations with phenotype and prognosis.

## 2. Materials and Methods

### 2.1. Study Subject

This study involved 172 female patients with cervical cancer from the Hospital of Lithuanian University of Health Sciences Kaunas Clinics. The research protocol was approved by the Kaunas Regional Biomedical Research Ethics Committee (protocol No. BE-2-10 and No. P1-BE-2-10/2014). Written informed consent was obtained from all the participants.

For this research, blood samples were collected from patients between October 2014 and August 2020. Patients were selected according to strict inclusion and exclusion criteria. The known clinicopathological characteristic data of patients, including age at the time of diagnosis, tumor-node-metastasis (TNM) classification, and tumor grade (G), were required for inclusion, as well as data about disease progression and death. Other malignancies, significant comorbidities, poor performance status, and incomplete medical documentation (the lack of information about 3 or more features) were considered as exclusion criteria. Clinicopathological characteristics were obtained from medical records with the help of oncologists. A flowchart presents patient selection (Supplementary Materials Figure S1).

### 2.2. DNA Extraction and Genotyping

Genomic DNA was isolated from peripheral white blood cells with the GeneJet Genomic DNA Purification Kit (Thermo Fisher Scientific Baltics, Vilnius, Lithuania; cat. no. K0721) according to the manufacturer’s instruction, and then stored at −20 °C until the PCR reaction was performed. Following a self-made protocol, *TIRAP* rs8177376, rs611953, rs3802814, and rs8177347 polymorphisms were analyzed with a polymerase chain reaction-restriction fragment length polymorphism (PCR-RFLP) assay. PCR amplification was carried out at a final volume of 25 μL for each sample. The reaction consisted of distilled water (dH_2_O), 2.5 µL of 10X DreamTaq Buffer (cat. no. B65), 0.38 µL of each primer (forward/reverse (20 µM); cat. no. 10629186), 0.5 µL of 10 mM dNTP Mix (cat. no. R1122), 0.13 µL of 5U/µL DreamTaq DNA Polymerase (cat. no. EP0701) (all reagents from Thermo Fisher Scientific Baltics, Vilnius, Lithuania), and 2 µL of template DNA (100 ng/µL). For each experiment, a negative control was used to check the contamination of components. DNA samples were amplified using specific primer sequences. After amplification, 10 μL of PCR products were digested by restriction enzymes (TasI (cat. no. ER1351), BseXI (cat. no. ER1452), Eco47I (cat. no. ER0311), or Eam1150I (cat. no. ER0241); Thermo Fisher Scientific Baltics, Vilnius, Lithuania) for 1–16 h in a total reaction volume of 15 μL for each sample. PCR and RFLP products were separated by 2 and 3% agarose gel electrophoresis, respectively, and then visualized by staining with 0.5 μg/mL of ethidium bromide under UV light. Table 1 shows the summarized PCR-RFLP reaction conditions.

### 2.3. Statistical Analysis

Statistical analysis was performed with the statistical software, Statistical Package for the Social Sciences (SPSS) version 27.0.1 (SPSS Inc., Chicago, IL, USA). Differences in genotype frequencies among the groups were evaluated by the Hardy–Weinberg equilibrium (HWE) using a chi-square test (*p >* 0.05). The associations between SNP genotypes (genotype model) or alleles (allelic model) and clinicopathological characteristics were analyzed using Pearson’s chi-square or Fisher’s exact tests. To estimate the impact of each SNP (increases or decreases the risk) on clinicopathological characteristics, the odds ratios (ORs) with a 95% confidence interval (CI) were calculated with univariate logistic regression. Two multivariate logistic regression models were used to estimate the adjusted ORs—Model No. 1 and Model No. 2. In Model No. 1, age at the time of diagnosis was considered as a potential covariate. Tumor size (T), lymph node involvement (N), and tumor grade (G) were included as additional covariates in Model No. 2. For multiple comparisons, a Bonferroni correction was applied. The difference was considered statistically significant when the *p*-value was less than 0.013 (Bonferroni-corrected *p* < 0.013).

For survival analysis, relationships between studied SNPs and overall survival (OS) and progression-free survival (PFS) were assessed. The time from the date of diagnosis till the event (local and systematic disease spread or last follow-up) was calculated as PFS; meanwhile, the time from the date of diagnosis until the date of death or last follow-up was considered as OS. Survival curves were generated using the Kaplan–Meier method and compared by a log-rank test. The hazard ratios (HRs) were calculated using univariate and multivariate Cox proportional hazards models. For multivariate logistic regression analysis, Model No. 1 (adjusted for age at the time of diagnosis) and Model No. 2 (adjusted for age at the time of diagnosis, T, N, G) were used. A *p*-value < 0.05 was considered statistically significant.

## 3. Results

### 3.1. The Distribution of Clinicopathological Characteristics, Genotypes, and Alleles of the Patients with Cervical Cancer

Table 2 presents the distribution of clinicopathological characteristics. Briefly, among the studied patients (*n* = 172), the mean age at the time of diagnosis was 55.4 ± 13.5 years. More than half of patients (*n* = 107; 62.2%) were older than 50 years old. Regarding clinicopathological characteristics, 110 (64.0%) patients had T1 or T2 tumors (smaller size), whereas the remaining patients had T3 or T4 tumors (larger size). Lymph node involvement was confirmed for 77 (44.8%) patients. Distant metastasis occurred only in 10 of 172 (5.8%) cases. A low (G1) or intermediate (G2) tumor grade was determined for the majority of patients (*n* = 125; 72.7%). During a follow-up, disease progression was confirmed for 51 patients (29.7%), whereas 121 (70.3%) cases were censored. A median PFS was 9.5 months (range 0–134). The death was confirmed for 40 (23.3%) patients after a median follow-up of 16 months (range 1–191). Of those who experienced progression, 36 patients died, all due to cancer-related death. Moreover, 4 patients died because of other reasons.

For every studied patient (*n* = 172), four *TIRAP* polymorphisms (rs8177376, rs611953, rs3802814, and rs8177374) were investigated. All SNPs were found to be in the Hardy–Weinberg equilibrium (*p* > 0.05). The frequency of alleles was as follows: 0.703 (T allele) and 0.297 (G allele) for rs8177376; 0.770 (G allele) and 0.230 (A allele) for rs611953; 0.872 (G allele) and 0.128 (A allele) for rs3802814; 0.863 (C allele) and 0.137 (T allele) for rs8177374. More detailed information about genotype and allele frequencies is presented in Supplementary Materials Table S1.

### 3.2. Association Analysis

In this study, the Pearson’s chi-square test was initially used to asses relationships between SNPs and clinicopathological features, including the fact of disease progression and patient death (Supplementary Materials Table S2). No statistically significant associations were confirmed (*p* > 0.013).

### 3.3. Logistic Regression Analysis

To evaluate whether SNPs increase or decrease the risk of selected clinicopathological characteristics, univariate logistic regression analysis was used. Multivariate logistic regression was only used in cases where the connections were statistically significant in univariate analysis. The results of univariate logistic regression analysis are shown in Supplementary Materials Table S3, whereas the results of multivariate logistic regression analysis are presented in Table 3.

In spite of the fact that rs8177376, rs611953, rs3802814, and rs8177374 were not found to be related to the analyzed cervical cancer features by Pearson’s chi-square analysis (*p* > 0.013), some statistically significant associations were discovered following logistic regression analysis.

According to the univariate logistic regression analysis, the rs8177376 T allele was associated with age at the time of diagnosis and tumor grade. Comparing T-allele carriers and non-carriers, it was shown that rs8177376 T-allele carriers had a higher probability to be diagnosed for cervical cancer at an older age (>50 years old) (OR = 1.768, 95% CI 1.274–2.453, *p* = 0.001). After adjustment for tumor size (T), lymph node involvement (N), and tumor grade (G), the association remained statistically significant (OR = 2.901, 95% Cl 1.750–4.808, *p* = 0.000). Additionally, the higher tumor grade (G3) was significantly less common in the carriers of the T allele of rs8177376 (OR = 0.354, 95% CI 0.247–0.508, *p* = 0.000) in comparison to T-allele non-carriers. This association remained significant after adjustment in the multivariate logistic regression analysis of Model No. 2 (OR = 0.424, 95% Cl 0.220–0.816, *p* = 0.010), even while this link was not significant in Model No. 1 (OR = 0.519, 95% Cl 0.307–0.875, *p* = 0.014).

Furthermore, univariate logistic regression demonstrated statistically significant associations between the rs611953 G allele and age at the time of diagnosis, as well as disease progression. Compared with non-carriers, the G allele of rs611953 was found to be more prevalent in the group of patients over 50 years old (OR = 1.721, 95% CI 1.256–2.360, *p* = 0.001). Even after adjusting for T, N, and G, this connection remained statistically significant in multivariate logistic regression analysis (OR = 3.258, 95% Cl 1.917–5.536, *p* = 0.000). It was also found that the carriers of the G allele had a lower probability of disease progression (OR = 0.419, 95% CI 0.300–0.585, *p* = 0.000) compared with G non-carriers. Based on multivariate logistic regression analysis, the association lost significance in both multivariate logistic regression models: Model No. 1 (OR = 0.555, 95% Cl 0.332–0.929, *p* = 0.025) and Model No. 2 (OR = 0.383, 95% Cl 0.110–1.341, *p* = 0.134). Thus, the results indicated that the role of other covariates is more important with regard to the impact the G allele of rs611953 has on cervical cancer.

Neither the rs3802814 nor rs8177374 polymorphisms demonstrated any significant relationships with the clinicopathological features under study in the genotype model based on univariate logistic regression analysis. Although the analysis in the allelic model revealed that the carriers of the G allele of rs3802814 had an approximately 2.4 times lower probability for disease progression (OR = 0.417, Cl 0.300–0.582, *p* = 0.000) compared with non-carriers, the assessment was controversial as only two patients had the AA genotype (T-allele non-carriers).

### 3.4. Survival Analysis

**Figure 1:**
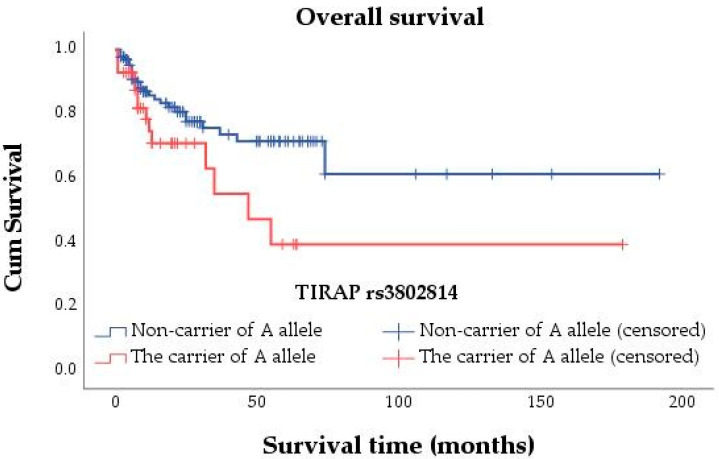
Kaplan–Meier survival curve for the association between the A allele of rs3802814 and OS.

**Figure 2:**
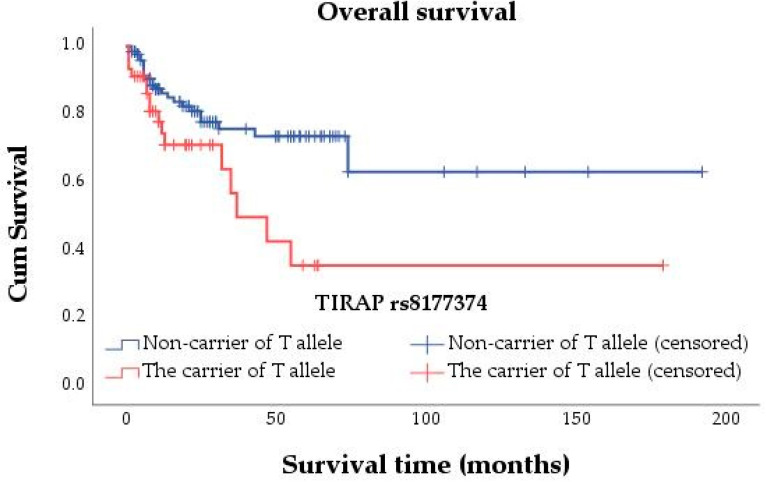
Kaplan–Meier survival curve for the association between the T allele of rs8177374 and OS.

We performed an analysis of the associations between *TIRAP* polymorphisms and PFS and OS using the log-rank test. Several associations were determined between rs3802814, rs8177374, and OS. The findings indicated that in the allelic model, OS was statistically associated with the A allele of rs3802814 (*p* = 0.038) and the T allele of rs8177374 (*p* = 0.012). In the genotype and allelic model, statistics for associations between OS and rs8177376 genotypes or the T allele, rs3802814 genotypes or the G allele, and rs8177374 genotypes or the C allele were not computed because all cases were censored. None of the tested SNPs were associated with PFS. The Kaplan–Meier method’s survival curves for statistically significant associations are shown in Figure 1 and Figure 2.

Using the univariate Cox regression analysis (Table 4), we determined that patients with the A allele of *TIRAP* rs3802814 had shorter OS (HR = 1.967, 95% CI 1.024–3.777, *p* = 0.042) compared with non-carriers.

The association remained statistically significant in Model No. 1 (HR = 1.967, 95% Cl 1.024–3.779, *p* = 0.042). When SNP was adjusted for age at the time of diagnosis, tumor size, lymph node involvement, and tumor grade (Model No. 2), no association of the A allele with OS (HR = 1.241, 95% Cl 0.627–2.458, *p* = 0.536) was detected, suggesting that other covariates are more important. The mean OS for A-allele carriers was 86.0 (95% CI 50.028–122.007) months, whereas the mean OS for A-allele non-carriers was 130.0 (95% CI 103.007–157.093) months.

Additionally, it was determined that the carriers of the T allele of *TIRAP* rs8177374 were more likely to have shorter OS (HR = 2.212, 95% CI 1.172–4.177, *p* = 0.014) in comparison to non-carriers (Table 4). Multivariate Cox regression analysis showed that association remained significant in Model No. 1 (HR = 2.212, 95% Cl 1.172–4.178, *p* = 0.014). The association loses significance if age at the time of diagnosis and other variables are adjusted (HR = 1.502, 95% Cl 0.776–2.907, *p* = 0.227). Thus, the T allele could not be used as an independent prognostic factor for survival. The mean OS in a group of T-allele carriers was 80.2 (95% CI 46.188–114.219) months, and 132.970 (95% CI 105.591–160.348) months in a group of T-allele non-carriers.

## 4. Discussion

The increasing effectiveness of the screening program and vaccination against HPV makes a significant contribution to the prevention and diagnosis of cervical cancer. Nevertheless, this disease remains one of the leading causes of death among women, especially in underdeveloped or developing countries (~85% of all cases) [3]. Persistent HPV infection is the main cause of cervical cancer. Studies have indicated that the detection and eradication of HPV highly depends on TLRs and the signaling pathways that they activate. However, the virus′s capacity to evade immune response makes prevention and detection challenging [2,3]. Although the mechanism is still unknown, it is clear that an abnormal inflammatory response is closely related to tumor pathogenesis [2,20].

TIRAP (or Mal) is one of the intracellular TLR signaling pathway molecules that controls immunological responses [8] and, therefore, modifications to TIRAP′s structure or functionality may have an impact on signal transduction. *TIRAP* polymorphisms have been studied in the context of lymphoma, glioma, and colorectal cancer, as we previously indicated; however, we were unable to locate any studies on cervical cancer. We were the first to examine the significance of the four loci for cervical cancer, rs8177376, rs611953, rs3802814, and rs8177374, as well as their correlation with clinicopathological traits and disease prognosis.

First of all, we investigated relationships between *TIRAP* rs8177376 and clinicopathological characteristics and clinical outcomes. Our research revealed that patients above the age of 50 had statistically higher T-allele prevalence. It was also found that the carriers of the T allele were less likely to have a higher tumor grade than non-carriers. The multivariate logistic regression analysis confirmed the statistical significance. Unfortunately, we cannot compare our results with others, because we did not find any studies analyzing associations of this polymorphism and clinicopathological characteristics. Nevertheless, reports have suggested that rs8177376 polymorphism is found in the *TIRAP* 3′-untranslated region, which is predicted to be involved in transcriptional regulation. It is believed that this SNP is located in a conserved binding site for miRNAs and may control gene expression at the post-transcriptional level [21]. Since data is not clear, it is difficult to assess the influence of SNP, but based on the results of our study, we suggest that the T allele could be associated with better prognosis. In survival analysis, we were unable to investigate genotypes or T allele and OS associations due to the lack of data, but other associations were non-significant. In the study by Stark et al. [22], an association between rs86177376 and OS was analyzed in patients with prostate cancer, although no significance was found. Therefore, we suggest that rs8177376 does not have a significant role in survival prognosis.

Similar results were observed between rs611953 and tumor features, as well as for rs8177376. The G allele showed a significant association with older age at the time of diagnosis. The association remained significant after multivariate logistic regression analysis. Moreover, we determined that carriers of the G allele had a lower probability of disease progression. The link, however, lost statistical significance when applying multivariate logistic regression, indicating that there may be other factors that are more significant for the investigated feature. Furthermore, rs611953 and PFS or OS did not appear to be significantly correlated in our results. Although we were unable to locate any research in the literature on this polymorphism in the context of cervical cancer, Klimosch et al. [16] did undertake a study on colon cancer. The rs611953 genotype was investigated in this study in relation to clinicopathological characteristics such as tumor size, lymph node involvement, the occurrence of distant metastases, and survival in colon cancer patients. It was demonstrated that the carriers of the GA genotype of rs611953 had a 1.9 times higher probability for distant metastases than homozygous carriers of the G allele. The association also remained significant after adjustment for age at diagnosis, T, and N in the multivariate analysis. Moreover, the OS was assessed; however, significance was not observed. Klimosch and colleagues also used a dominant model (GA + AA vs. GG genotype) which also showed that patients with the GA + AA genotype were more likely to have distant metastasis (OR = 1.92, Cl 95% 1.26–2.95, *p* = 0.003) and shorter OS. Based on the results, we believe that the G allele could be related to a better prognosis.

In this study we did not find any significant associations between rs3802814 and clinicopathological characteristics in the genotype model. In the allelic model it was observed that the G allele was associated with a lower risk of disease progression; however, the evaluation of the result is quite conflicting, because only two patients did not have the G allele. More studies are required to confirm or deny this observation. Moreover, we found a statistically significant association between the A allele of rs3802814 and OS. The results from the survival analysis showed that the A allele was associated with shorter OS in the univariate Cox regression analysis and after adjustment by age at the time of diagnosis, but no significance was found when more additional covariates were included inthe analysis. As a result, the significance of other factors should be assessed. Unfortunately, we did not find any study investigating associations between this SNP and clinicopathological characteristics or clinical outcomes in patients with cancer in general.

Among the many SNPs in *TIRAP*, rs8177374 is the most commonly researched polymorphism. The genetic change includes a cytosine to thymine transition resulting in the substitution from serine (Ser) to leucine (Leu) at position 180 (S180L). This may impair the TIRAP function and alter NF-κBsignaling, which would result in reduced production of pro-inflammatory cytokines and protection against excessive inflammation [6,7]. However, the significance of this polymorphism for disease pathogenesis is contradictory. On the one hand, the TT genotype of the rs8177374 polymorphism (Leu/Leu phenotype; L180L) may lead to increased risk of infectious and other diseases due to a decreased immune response. On the other hand, the CC genotype (Ser/Ser; S180S) induces a more intense signaling and pro-inflammatory cytokine response. Meanwhile, CT (Ser/Leu; S180L) is considered an optimal and protective genotype in disease pathogenesis because studies have shown that patients carrying one copy of the T allele are less susceptible to infections [6]. The CT genotype has been linked to pneumococcal disease, bacteremia, malaria, tuberculosis [23], and tuberculosis meningitis [11]. In our study, a statistically significant correlation was only observed in survival analysis. Following the univariate logistic regression analysis and adjusting for age at diagnosis, it was discovered that the T allele was associated with a worse OS. However, Model No. 2 lost significance, demonstrating the impact of other covariates. The effects of rs8177374 on patients with symptomatic multiple myeloma were examined by Bagratuni et al. [24]. The study sample’s genotype distribution was the same as ours: CC was present in 74%, CT in 25%, and TT in 1%. They showed that PFS and OS were considerably shorter in *TIRAP* SNP carriers (*p* < 0.05) even after multivariate Cox regression analysis. Thus, we hypothesize that rs8177374 could be associated with worse prognosis of survival; however, more detailed studies are needed.

Several limitations were observed in this study. Firstly, we aimed to analyze the associations of SNPs with tumor characteristics and clinical outcomes. Therefore, we did not analyze the control group and did not assess the impact of studied polymorphisms on the risk of cervical cancer. Secondly, because of multiple comparisons, we used Bonferroni corrections and a *p*-value less than 0.013; therefore, some really important associations may have been considered non-significant. Thirdly, our sample size was limited; thus, a larger group of patients with cervical cancer is required to confirm the results that were obtained.

## 5. Conclusions

In conclusion, we identified that *TIRAP* polymorphisms increase the probability for an older age at the time of diagnosis (rs8177376 and rs611953) and lower tumor grade (rs8177376) in cervical cancer. However, additional thorough and large-scale studies are required to test the relationships and validate the findings of our study before using the polymorphisms as biomarkers in the prognosis of cervical cancer.

## Figures and Tables

**Table 1:** Reaction conditions for PCR-RFLP assay.

SNP	PCR				RFLP		
	Primer Sequences	Annealing Temperature (°C)	Number of Cycles	Fragment Size of PCR Product (bp)	Restriction Enzyme	Incubation Temperature (°C)	Fragment Size (bp)
rs8177376	F: 5′-GGTTTGGGAG	58.0	30	245	TasI (Tsp509I) ^3^	65	T: 158, 87 G: 245
	GTGTGACAAC-3′ ^1^						
	R: 5′-ATGGTCTTCTT						
	AGGGAGCCC-3′ ^1^						
rs611953	F: 5′-GTGACAACGC	57.6	30	223	BseXI (BbvI) ^3^	65	A: 223 G: 170, 53
	TGTGATTGGT-3′ ^1^						
	R: 5′-TAGGGAGCCC						
	ACAGTAATGG-3′ ^1^						
rs3802814	F: 5′-AGCCTCAGCT	58.4	30	153	Eco47I (AvaII) ^3^	37	G: 126, 27 A: 153
	CAGTCACGTC-3′ ^1^						
	R: 5′-GCTGCCTTCCA						
	AGTAGGAGA-3′ ^1^						
rs8177374	F: 5′-AGTGCTGTACC	64.4	40	161	Eam1150I (AhdI) ^3^	37	C: 141, 20 T: 161
	ATCGACCTGCTG-3′ ^2^						
	R: 5′-TTCCCCTTCTCCC						
	TCCTGTAGTAG-3′ ^2^						

F—forward primer; R—reverse primer; ^1^—primer sequences were generated using https://primer3.ut.ee (accessed on 23 September 2020); ^2^—primer sequences were described by Zhang et al. [19]; ^3^—the restriction enzymes were selected using http://nc2.neb.com/NEBcutter2/program (accessed on 25 September 2020).

**Table 2:** Clinicopathological characteristics of the patients with cervical cancer (*n* = 172).

Clinicopathological Characteristics	Subgroups	*n* (%)
Age group	≤50 years old	65 (37.8)
	>50 years old	107 (62.2)
Tumor size (T)	T1 + T2	110 (64.0)
	T3 + T4	62 (36.0)
Lymph node involvement (N)	N0	95 (55.2)
	N1	77 (44.8)
Metastasis (M)	M0	162 (94.2)
	M1	10 (5.8)
Tumor grade (G)	G1 + G2	125 (72.7)
	G3 X^1^	45 (26.2) 2 (1.1)
Presence of disease progression	No	121 (70.3)
	Yes	51 (29.7)
Death	No	132 (76.7)
	Yes	40 (23.3)

X^1^—missing data; T—tumor size; T1 + T2—smaller tumor size; T3 + T4—larger tumor size; N—lymph node involvement; N0—no regional lymph node metastasis; N1—regional lymph node metastasis; M—metastasis; M0—no distant metastasis; M1—distant metastasis; G—tumor grade; G1—low grade (well-differentiated); G2—intermediate grade (moderately differentiated); G3—high grade (poorly differentiated).

**Table 3:** Multivariate logistic regression analysis for associations between *TIRAP* rs8177376, rs611953 and clinicopathological characteristics.

SNP	Dependent	Covariates	Multivariate Logistic Regression Analysis					
			Model No. 2			Model No. 3		
			OR	95% Cl	*p*	OR	95% Cl	*p*
rs8177376	Older age at the time of diagnosis (>50 years old)	Carrier of T allele vs. non-carrier	-	-	-	2.901	1.750–4.808	** 0.000**
		Age group	-	-	-	-	-	-
		T (T3 + T4 vs. T1 + T2)				1.777	0.831–3.802	0.138
		N (positive vs. negative)				0.288	0.143–0.581	0.000
		G (G3 vs. G1 + G2)				0.659	0.320–1.357	0.258
	G3 tumor grade	Carrier of T allele vs. non-carrier	0.519	0.307–0.875	0.014	0.424	0.220–0.816	**0.010**
		Age group		0.276–1.028	0.060	0.501	0.255–0.983	0.045
		T (T3 + T4 vs. T1 + T2)				2.094	0.976–4.496	0.058
		N (positive vs. negative)				0.869	0.426–1.770	0.698
		G (G3 vs. G1 + G2)	-	-	-	-	-	-
rs611953	Older age at the time of diagnosis (>50 years old)	Carrier of G allele vs. non-carrier	-	-	-	3.258	1.917–5.536	**0.000**
		Age group	-	-	-	-	-	-
		T (T3 + T4 vs. T1 + T2)				1.799	0.840–3.852	0.131
		N (positive vs. negative)				0.242	0.117–0.501	0.000
		G (G3 vs. G1 + G2)				0.581	0.276–1.223	0.153
	Disease progression	Carrier of G allele vs. non-carrier	0.555	0.332–0.929	0.025	0.383	0.110–1.341	0.134
		Age group		0.325–1.224	0.173	0.574	0.267–1.234	0.155
		T (T3 + T4 vs. T1 + T2)				7.587	3.367–17.092	0.000
		N (positive vs. negative)				0.731	0.321–1.667	0.457
		G (G3 vs. G1 + G2)				0.856	0.516–1.420	0.547

OR—odds ratio; Cl—confidence interval; vs.—versus; Age group (age at the time of diagnosis): >50 years old vs. ≤50 years old; T—tumor size: T3 + T4 (larger) vs. T1 + T2 (smaller); N—lymph node involvement: N1 (regional lymph node metastasis) vs. N0 (no regional lymph node metastasis); G—tumor grade: G3 (high grade (poorly differentiated)) vs. G1 + G2 (low grade (well-differentiated) or intermediate grade (moderately differentiated), respectively). Statistically significant *p* values are marked in bold (Bonferroni-corrected *p* < 0.013).

**Table 4:** Univariate and multivariate Cox regression analysis between the rs3802814 A allele and rs8177374 T allele and OS.

Variables	Univariate			Multivariate (Model No. 1)			Multivariate (Model No. 2)		
	HR	95% Cl	*p*	HR	95% Cl	*p*	HR	95% Cl	*p*
rs3802814 A allele Age group T N G	1.967	1.024–3.777	**0.042**	1.967	1.024–3.779	**0.042**	1.241	0.627–2.458	0.536
				1.019	0.537–1.934	0.955	1.442	0.727–2.861	0.295
							7.897	3.383–18.433	0.000
							1.667 0.685	0.793–3.502 0.336–1.393	0.177 0.296
rs8177374 T allele Age group T N G	2.212	1.172–4.177	**0.014**	2.212	1.172–4.178	**0.014**	1.502	0.776–2.907	0.227
				0.995	0.524–1.889	0.988	1.451	0.729–2.887	0.289
							7.743	3.327–18.025	0.000
							1.639	0.776–3.460	0.195
							0.681	0.335–1.386	0.289

HR—hazard ratio; Cl—confidence interval; OR—odds ratio; Age group (age at the time of diagnosis): >50 years old vs. ≤50 years old; T—tumor size: T3 + T4 (larger) vs. T1 + T2 (smaller); N—lymph node involvement: N1 (regional lymph node metastasis) vs. N0 (no regional lymph node metastasis); G—tumor grade: G3 (high grade (poorly differentiated)) vs. G1 + G2 (low grade (well-differentiated) or intermediate grade (moderately differentiated), respectively). Statistically significant *p* values are marked in bold (*p* < 0.05).

## Data Availability

The data presented in this study are available from the corresponding author on reasonable request.

## Supplementary Materials

The following supporting information can be downloaded at: https://www.mdpi.com/article/10.3390/genes13081365/s1, Figure S1: Patients selection; Table S1: The distribution of genotypes and alleles of TIRAP rs8177376, rs611953, rs3802814, rs8177374 polymorphisms in patients with cervical cancer (*n* = 172); Table S2: The results by Pearsons’ Chi-square test in association analysis between alleles of TIRAP rs8177376, rs611953, rs3802814, rs8177374 and clinicopathological characteristics (*n* = 172); Table S3: Univariate logistic regression analysis between TIRAP polymorphisms and clinicopathological characteristics, disease progression and death (*n* = 172).
