# Supplementary figures and images for: TIRAP Rs8177376, Rs611953, Rs3802814, and Rs8177374 Polymorphisms and Their Association with Cervical Cancer Phenotype and Prognosis
Source: Genes (Basel). 2022 Jul 29;13(8):1365. doi: 10.3390/genes13081365 (PMC9407394; doi:10.3390/genes13081365)

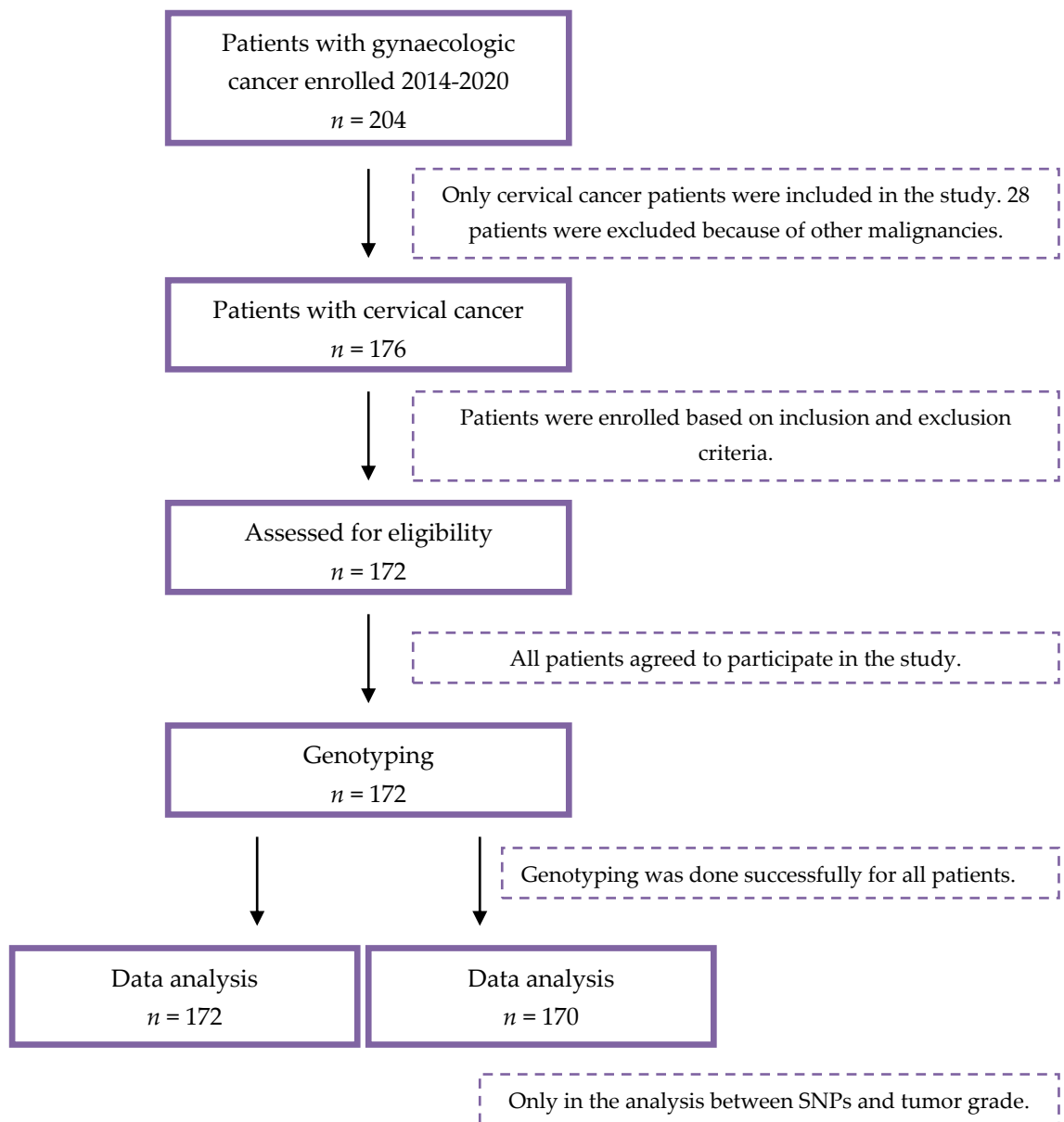

Supplementary Material Figure S1. Patients selection

Supplement: Supplementary file 1 [file genes-13-01365-s001.zip › Supplementary Material Figure S1.pdf]
